# The Tariff on Manufactured Teeth

**Published:** 1891-02

**Authors:** 


### Editorial.

### THE TARIFF ON MANUFACTURED TEETH.

When the so-called McKinley bill was passed by both houses of Congress and signed by the President, it is questionable whether the dental profession at large had any idea that it touched their interests, only as it indirectly affected the great mass of the people. It was therefore doubtless with surprise that they discovered that they were to be subjected to the unpleasant effect of a prohibitory clause, which must appear to any reflecting mind as being wholly unnecessary and one in direct opposition to the best interests of operators and patients.

On examination of the official "Comparison of the Custom Laws of 1883 with the New Law of 1890," it is found, under the heading "Schedule N., Sundries," that "Items specially provided for under the old law, which will be classified under the new law according to the component material of chief value," there is given, among other articles, "Teeth manufactured, twenty per cent. *ad valorem*" (old law). As porcelain is the "component part" of teeth, an examination of "Schedule B" gives us the new law. "China, porcelain, parian, etc., . . . not specially provided for in this act, . . . if not ornamented or decorated, *fifty-five per centum ad valorem.*"

It is not the purpose of this journal to question the policy of protection in any sense, as that would be beyond its province; but when a blow is aimed directly, as this does, at an important part of our profession,—mechanical dentistry,—it is certainly not only our duty, but that of every individual connected with our calling, to protest, and ask for its repeal. The twenty per cent. *ad valorem* was ample protection to the manufacturing interests engaged in this specialty, if protection were needed at all.

The production of artificial teeth has been, from the earliest periods of the manufacture, almost exclusively confined to the United States. The product has occupied, very justly, a pre-eminent place in the work of the world, and has never stood in danger of competition. In no country of Europe has there ever been an approach to the results obtained here, and only in one case has any manufacturing establishment on the other side of the ocean been able to compete in Europe with those specially engaged in this work in the United States. This exhibit is a remarkable one in view of the extraordinary development of porcelain manufacture in other lines in Europe.

The few engaged in this manufacture in this country have grown wealthy, as they practically enjoyed for many years the monopoly of the markets of the world, and, as far as this country is concerned, the importation of foreign manufactured teeth has been in the past of insignificant proportions.

A demand has, however, grown up in recent years for the product of an English manufacturer, for the reason that these teeth possessed some advantages for certain kinds of dentures, especially that of "bridge-work." This demand evidently began to assume disagreeable proportions, or no one would have heard of an increase of duty.

The situation is now such that it will be impossible to procure these teeth without great difficulty and serious additional expense. The latter is not of so much moment as the fact that they will probably not be obtainable here at any price.

Now, it may be asked, why this increase of duty? Is the industry one that requires protection? Have the gentlemen who have been engaged in this manufacture for the past half-century not realized an equivalent for the capital invested? Have they at any time been in competition with the cheap labor of Europe? These questions, and more, require answers from those financially interested. We have no information to lead to the supposition that undue influence has been used to secure this additional charge; but it is singular that the Committee of Congress should have decided to increase the duty thirty-five per cent. without solicitation or knowledge. It would be interesting to know the reasons for this great increase.

As a profession, we have nothing to do with the political aspects of this question; but it does seem as though active efforts should be made to secure the repeal of this obnoxious clause, for which not a shadow of reason exists.

